# Clinical value of controlling nutritional status scores in patients with hepatocellular carcinoma

**DOI:** 10.3389/fnut.2025.1598463

**Published:** 2025-06-30

**Authors:** Rongqiang Liu, Lei Ji, Chenxuan Zhang, Jing Ye, Xinyi Li, Wangbin Ma, Jia Yu, Weixing Wang

**Affiliations:** Department of Hepatobiliary Surgery, Renmin Hospital of Wuhan University, Wuhan, China

**Keywords:** controlling nutritional status score, hepatocellular carcinoma, prognosis, survival, meta-analysis

## Abstract

**Background:**

Numerous studies have highlighted the significance of controlling nutritional status (CONUT) score in the prognosis of patients with hepatocellular carcinoma (HCC). However, the precise role of the CONUT scor in predicting HCC prognosis remains unclear. This study aimed to investigate the prognostic significance of the CONUT scores in patients with HCC through an updated meta-analysis.

**Methods:**

Three internationally recognized authoritative databases (PubMed, Web of Science and Embase) were searched. The research focused on the relationship between CONUT score and prognosis in patients with HCC. The deadline for literature search was October 23, 2024. In this study, hazard ratios (HRs) and odds ratios (ORs) were used as the primary statistical indicators for pooled analysis. All calculated HRs and ORs were accompanied by 95% confidence intervals (95% CIs). Data analyses were performed using STATA software, version 12.0.

**Results:**

A total of 19 relevant studies encompassing 7,963 patients were included in this meta-analysis. The CONUT score was significantly associated with unfavorable survival outcomes, including overall survival (OS) (HR: 1.58, 95% CI: 1.33–1.87), disease-free survival (DFS) (HR: 2.06, 95% CI: 1.34–3.18), recurrence-free survival (RFS) (HR: 1.48, 95% CI: 1.09–2.00), and progression-free survival (PFS) (HR: 1.93, 95% CI: 1.24–2.99). Subgroup analysis further confirmed the prognostic value of the CONUT score. Moreover, high CONUT score was strongly associated with tumor differentiation (poor vs. moderate/well) (OR: 1.32, 95% CI: 1.16–2.35) and tumor number (solitary vs. multiple) (OR: 1.25, 95% CI: 1.19–1.84).

**Conclusion:**

HCC patients with high CONUT scores usually face a worse survival prognosis. The CONUT score may be a valid prognostic indicator for patients with HCC.

## Introduction

Liver cancer is one of the most common and deadly cancers worldwide. Its high morbidity and mortality rates pose a major global health challenge. The number of new cases continues to rise each year, and the trend that is expected to persist as the population ages and the prevalence of chronic diseases increases (1). The early symptoms of liver cancer are often subtle, leading to many patients being diagnosed at an advanced stage, which results in poor treatment outcomes and low survival rates. Therefore, the prevention and early diagnosis of liver cancer are among the current priorities in public health. Hepatocellular carcinoma (HCC) is the most prevalent form of primary liver cancer. Hepatitis B virus (HBV) infection is the most significant risk factor for HCC, with approximately 50% of global HCC cases attributed to HBV. In developed countries, hepatitis C virus (HCV) infection is a leading cause of HCC, accounting for approximately 30% of HCC cases. The persistent liver inflammation and damage caused by these infections promote abnormal cell proliferation and carcinogenesis. Patients with long-term chronic hepatitis are at significantly higher risk for developing HCC (2). In addition, the incidence of HCC associated with metabolically associated non-alcoholic steatohepatitis is increasing, especially in Western countries (3). For HCC, radical resection is currently the only treatment that offers a chance of cure (4). However, early symptoms of HCC are often insidious, resulting in many patients being diagnosed only when the disease has progressed to an advanced stage. By this time, the tumor may have extended beyond the liver or be accompanied by severe liver dysfunction, making treatment options even more limited (5). Therefore, stratification and personalized treatment are crucial for patients with HCC.

The controlling nutritional status (CONUT) score was introduced in 2012 as a method for assessing the patient’s nutritional status. Due to its simplicity, cost-effectiveness, and clinical applicability, the CONUT score has been widely employed in the nutritional assessment and prognostic prediction of various diseases (6). It combines three important biomarkers: serum albumin, cholesterol levels, and lymphocyte count. These indicators reflect the patient’s nutritional status, immune function, and systemic inflammatory status. Detailed information on the CONUT scoring system is summarized in Table 1. Several cumulative studies have indicated that the CONUT score is not only effective in assessing the nutritional status of patients but also serves as a reliable prognostic marker for diverse tumors, including hematologic and solid tumors (7–10).

**Table 1:** The controlling nutritional status scoring system.

Parameters	Degree			
	Normal	Light	Moderate	Severe
Serum albumin (g/dL)	≥3.5	3.0–3.49	2.50–2.99	<2.50
Score	0	2	4	6
Total lymphocyte count (/mm^3^)	≥1,600	1,200–1,599	800–1,199	<800
Score	0	1	2	3
Total cholesterol (mg/dL)	≥180	140–179	100–139	<100
Score	0	1	2	3
CONUT score	0–1	2–4	5–8	9–12

Unlike traditional prognostic tools such as the BCLC staging system or Child-Pugh score, which primarily focus on tumor burden and liver function, the CONUT score provides an objective assessment of the patient’s nutritional and immunological status based on serum albumin, total cholesterol, and lymphocyte count. Malnutrition and immune suppression are common in HCC patients, especially those with underlying cirrhosis, and can significantly influence treatment tolerance and prognosis (11). Moreover, it could guide therapeutic decisions, such as the need for nutritional support, immunonutritional interventions, or closer monitoring in patients undergoing surgery, locoregional therapy, or systemic treatment (12). Therefore, exploring the clinical utility of CONUT may help refine risk stratification and optimize individualized treatment strategies beyond what is possible with traditional tools alone.

Numerous academic studies have explored the relationship between the CONUT score and HCC. A meta-analysis of five small-sample studies suggested that the CONUT score may serve as a prognostic indicator for HCC (13). However, as further research is conducted, the prognostic value of the CONUT score in HCC remains controversial. Some studies have failed to establish a significant association between the two, indicating that their predictive value may be influenced by confounding factors (14–18). Consequently, a more systematic and comprehensive analysis is necessary to clarify the predictive value of the CONUT score in HCC patients. This study implemented a systematic review and updated meta-analysis to assess whether the CONUT score can be used as a reliable prognostic indicator for HCC patients, aiming to resolve discrepancies in existing studies and provide more definitive evidence.

## Materials and methods

### Search strategy

To comprehensively evaluate the relationship between the CONUT score and the prognosis of HCC patients, this study conducted an extensive literature search, covering major databases such as PubMed, Web of Science, and EMBASE. Relevant articles were screened based on specific keywords and topics to ensure coverage of the latest research findings and to avoid omission of important literature. The deadline for all searches was October 23, 2024. The following search terms were used: "controlling nutritional status" OR "CONUT’ OR "controlling nutritional status score" OR "CONUT score" AND "hepatocellular carcinoma" OR "hepatic carcinoma" OR "hepatoma" OR "liver cancer" OR "HCC" AND "prognosis" OR "survival OR "prognostic’ OR "outcome". The language was not restricted. In addition to the initial database search, a manual search was conducted in this study, which included a thorough examination of the references from the included studies. This supplementary search strategy enabled a more comprehensive integration of relevant literature and helped minimize potential omissions and bias.

### Inclusion and exclusion criteria

The literature included in this study had to meet the following criteria: (1) investigation of the association between the CONUT score and survival outcomes in patients with HCC; (2) provision of adequate data to calculate hazard ratios (HRs) and 95% confidence intervals (CIs). The exclusion criteria were as follows: (1) reviews, case reports, letters, commentaries, and conference abstracts; (2) studies lacking sufficient data; and (3) studies with duplicated data.

### Data extraction and quality assessment

Two independent researchers reviewed each included article and extracted data from the included studies. When discrepancies or uncertainties arose during the data extraction process, a third independent researcher was consulted to arbitrate and ensure the accuracy and consistency of the final data. A standardized data collection form was used to extract the following information: the first author’s name, year of publication, country, study design, sample size, type of analysis, and survival outcomes. All included studies were assessed for quality using the Newcastle-Ottawa Scale (NOS). In this study, a score greater than 6 was considered indicative of high-quality studies, ensuring that the included literature met high methodological standards and demonstrated reliable results (19).

### Statistical analysis

In the study, all data analyses were conducted using STATA version 12.0 (Stata Corporation, College Station, TX, USA). To assess heterogeneity among the included studies, we utilized the I^2^ statistic. When the I^2^ value was <50%, it indicated low heterogeneity between studies, and a fixed-effect model was applied for data analysis. Conversely, when the I^2^ value was >50%, a random-effects model was used to integrate the data more appropriately. To further explore potential sources of heterogeneity, we performed subgroup analyses by grouping studies based on different characteristics. Additionally, sensitivity analyses were conducted to evaluate the stability and reliability of the meta-analysis results. The Begg test, Egger test, and trim-and-fill method were used to assess the presence of publication bias and its impact on the results (20). *p*-value less than 0.05 was regarded as a statistically significant threshold.

## Results

### Search results

**Figure 1:**
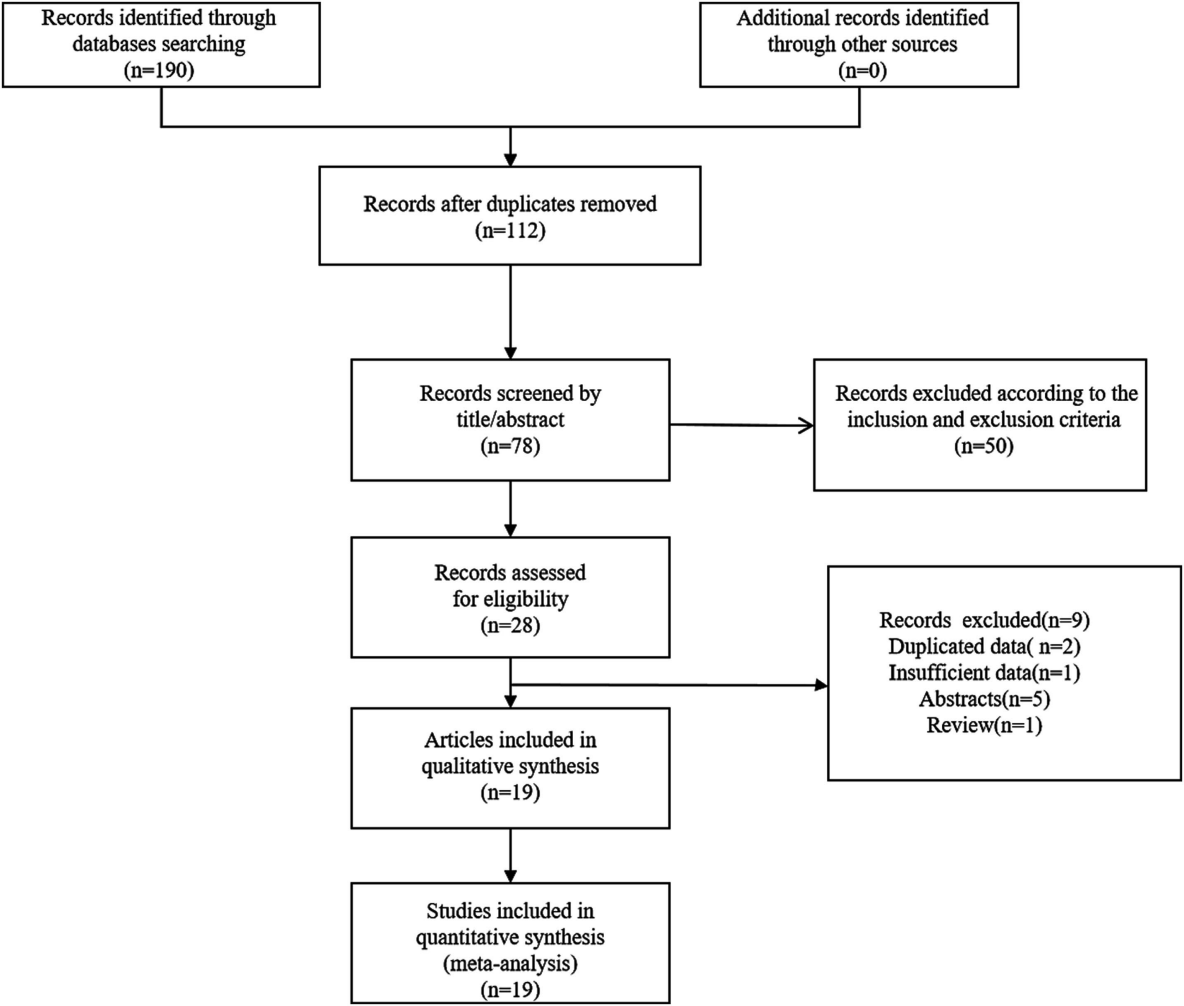
Flow chart of study search and selection.

We found 190 relevant articles by searching relevant databases and excluding 112 duplicates. After browsing titles and abstracts, 78 articles were excluded. This meta-analysis incorporated 19 relevant studies, encompassing a total of 7,963 patients (14–18, 21–34). The detailed steps and methods of the screening process were illustrated in Figure 1.

### Study characteristics

Table 2 summarized the clinical characteristics of the included studies. Among the 19 studies included, 18 were published in English and one in Chinese. These studies spanned multiple countries, with 10 conducted in China, 7 in Japan, and 1 each in Germany and Turkey. The survival outcomes reported in these studies primarily encompassed multiple dimensions, including overall survival (OS), disease-free survival (DFS), recurrence-free survival (RFS), and progression-free survival (PFS). Regarding statistical analysis methods, 14 studies employed multivariate analyses, while 5 utilized univariate analyses. Additionally, the quality assessment of all included studies exceeded 5 with a mean score of 6.8, indicating the high quality of these studies.

**Table 2:** Basic information of the included articles.

Study	Year	Country	Study type	Sample	Treatment method	Cut off value	Analysis type	Survival analysis	NOS score
Chen	2020	China	R	325	RFA	>5	MVA	OS, RFS	7
Chen	2022	China	R	228	TACE	≥4	MVA	OS, PFS	7
Harimoto	2018	Japan	R	2,461	Surgery	≥4	MVA	OS, RFS	8
Lin	2020	China	R	380	Surgery	≥2	MVA	OS, RFS	7
Matsumoto	2022	Japan	R	493	Surgery	≥2	MVA	OS	6
Müller	2021	Germany	R	237	TACE	>3	MVA	OS	6
Qian	2022	China	R	661	Surgery	>3	MVA	OS, RFS	7
Shimose	2020	Japan	R	164	Lenvatinib	≥5	MVA	OS	6
Takagi	2017	Japan	R	295	Surgery	≥5	MVA	OS, RFS	7
Tamai	2022	Japan	R	181	Surgery	≥3	MVA	OS, PFS	7
Tsunematsu	2020	Japan	R	246	Surgery	≥4	MVA	OS, DFS	7
Wang	2019	China	R	209	Surgery	≥3	MVA	OS, RFS	7
Yang	2020	China	R	403	RFA	≥5	MVA	OS, DFS	8
Pravisani	2020	Italy	R	280	Surgery	NA	UVA	OS, RFS	8
Deng	2024	China	R	284	TACE	≥4	MVA	OS, PFS	8
Peng	2021	China	R	547	Surgery	≥3	UVA	OS, RFS	7
Fujio	2022	Japan	R	64	Surgery	≥3	UVA	OS, RFS	6
Chen	2022	China	R	20	Immunotherapy plus targeted therapy	>2	UVA	PFS	5
Wang	2019	China	R	470	Surgery	>3	UVA	OS	6

R, retrospective; OS, overall survival; DFS, disease-free survival; RFS, recurrence free survival; PFS, progression-free survival; MVA: multivariable analysis; UVA: univariable analysis; NOS score, Newcastle-Ottawa Scale score; RFA, Radiofrequency Ablation; TACE, transarterial chemoembolization; NA, not available.

### Impact of CONUT score on OS

**Figure 2:**
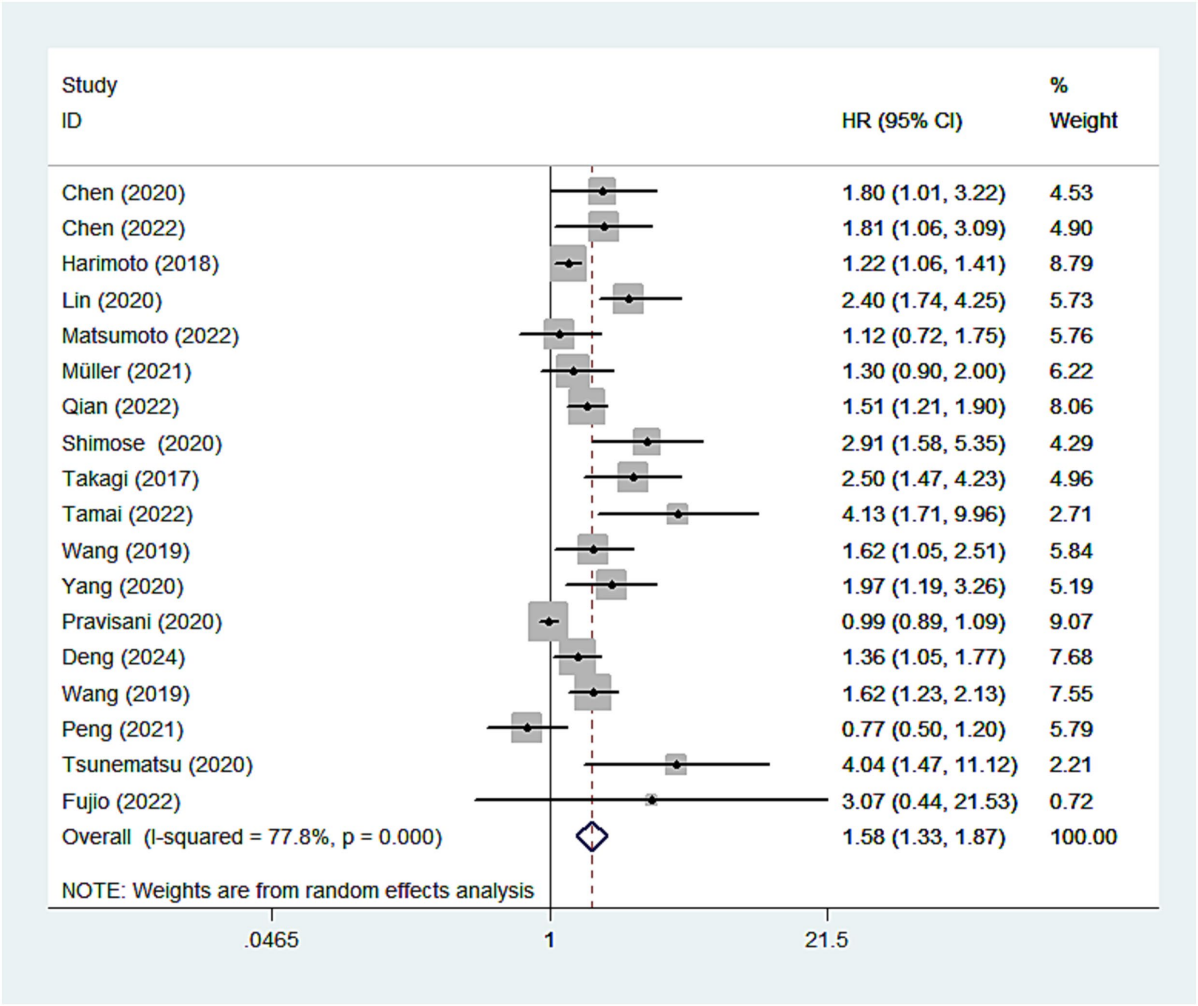
Forest plots of the association between high CONUT score and OS. CONUT, controlling nutritional status; OS, overall survival.

In the meta-analysis, 18 studies investigated the relationship between the CONUT score and OS in patients with HCC. Given the significant heterogeneity among the studies, the pooled analysis was conducted using a random-effects model (I^2^ = 78%). The results demonstrated a significant negative correlation between the CONUT score and overall survival in HCC patients. Specifically, patients with higher CONUT scores exhibited poorer overall survival, suggesting that the CONUT score may serve as a valuable indicator for prognostic assessment in HCC patients (HR: 1.58, 95%CI: 1.33–1.87) (Figure 2).

### Subgroup analysis and meta-regression for OS

In the meta-analysis, subgroup analysis was analyzed based on country, treatment, sample size and analysis type (Table 3). In the subgroups of China (HR: 1.53, 95%CI: 1.36–1.72) and Japan (HR: 2.30, 95% CI: 1.48–3.56), high CONUT score were significantly associated with poorer OS. High CONUT score was also linked to poorer prognosis in both surgical (HR: 1.57, 95%CI: 1.26–1.96) and non-surgical groups (HR: 1.58, 95% CI: 1.33–1.88), as well as in sample sizes greater than or less than 300. Within the multivariate analysis group, a high CONUT score demonstrated strong predictive ability (HR: 1.75, 95% CI: 1.47–2.10). Subgroup analysis indicated that countries and treatment methods may be the sources of heterogeneity. Variations in patient populations across countries and differing treatment modalities may contribute to survival disparities, thereby influencing the relationship between CONUT scores and OS. Furthermore, meta-regression found that the analysis type contributed to heterogeneity (*p* = 0.048).

**Table 3:** Subgroup analysis and meta-regression for OS.

Factors	Studies	HR (95%)	OS impact	*p*	Heterogeneity		Meta-regression			
					I^2^	*p*	Model	Tau^2^	Adj R^2^ (%)	*p*
OS										
Country								0.108	−2.8	0.6
China	9	1.53 (1.36–1.72)	↑✓	<0.01	49.1	0.047	Fixed			
Japan	8	2.30 (1.48–3.56)	↑✓	<0.01	76.5	<0.01	Random			
Italy	1	0.985 (0.892–1.088)	**×**							
Germany	1	1.30 (0.90–2.0)	**×**							
Treatment method								0.112	−6.64	0.723
Non-surgery	6	1.58 (1.33–1.88)	↑✓	<0.01	30.4	0.208	Fixed			
Surgery	13	1.57 (1.26–1.96)	↑✓	<0.01	81.8	<0.01	Random			
Sample size								0.12	−14.2	0.25
>300	8	1.44 (1.18–1.76)	↑✓	<0.01	66.3	0.004	Random			
<300	11	1.93 (1.41–2.65)	↑✓		81.6	<0.01	Random			
Analysis type								0.012	61.09	0.048
MV	15	1.75 (1.47–2.10)	↑✓	<0.01	63	0.001	Random			
UV	4	1.13 (0.78–1.63)	**×**	0.509	78.7	0.003	Random			
RFS										
Country								0.009	74.69	0.71
China	5	1.37 (1.20–1.56)	↑✓	0.161	39.1	0.021	Random			
Japan	3	1.28 (1.12–1.45)	↑✓	0.208	36.3	0.063	Random			
Italy	1	0.94 (0.82–1.07)	**×**							
Treatment method								0.0328	8.57	0.287
Non-surgery	1	1.73 (1.16–2.58)	↑✓							
Surgery	8	1.25 (1.05–1.47)	↑✓	0.01	70.7	0.001	Random			
Sample size										
>300	5	1.28 (1.16–1.41)	↑✓	<0.01	42.5	0.138	Fixed	0.044	−24.33	0.961
<300	4	1.33 (1.3–12.00)	↑✓	0.001	80	0.231	Random			
Analysis type								0.003	91.28	0.006
MV	6	1.35 (1.23–1.49)	↑✓	<0.01	8.1	0.365	Fixed			
UV	3	0.94 (0.83–1.06)	**×**	0.318	0	0.504	Random			

R, retrospective; OS, overall survival; RFS, recurrence free survival; MVA: multivariable analysis; UVA: univariable analysis; ↑ indicates worse survival with high CONUT scores; ✓ = statistically significant (*p* < 0.05); **×** = not statistically significant.

### Impact of CONUT score on DFS/RFS/PFS

**Figure 3:**
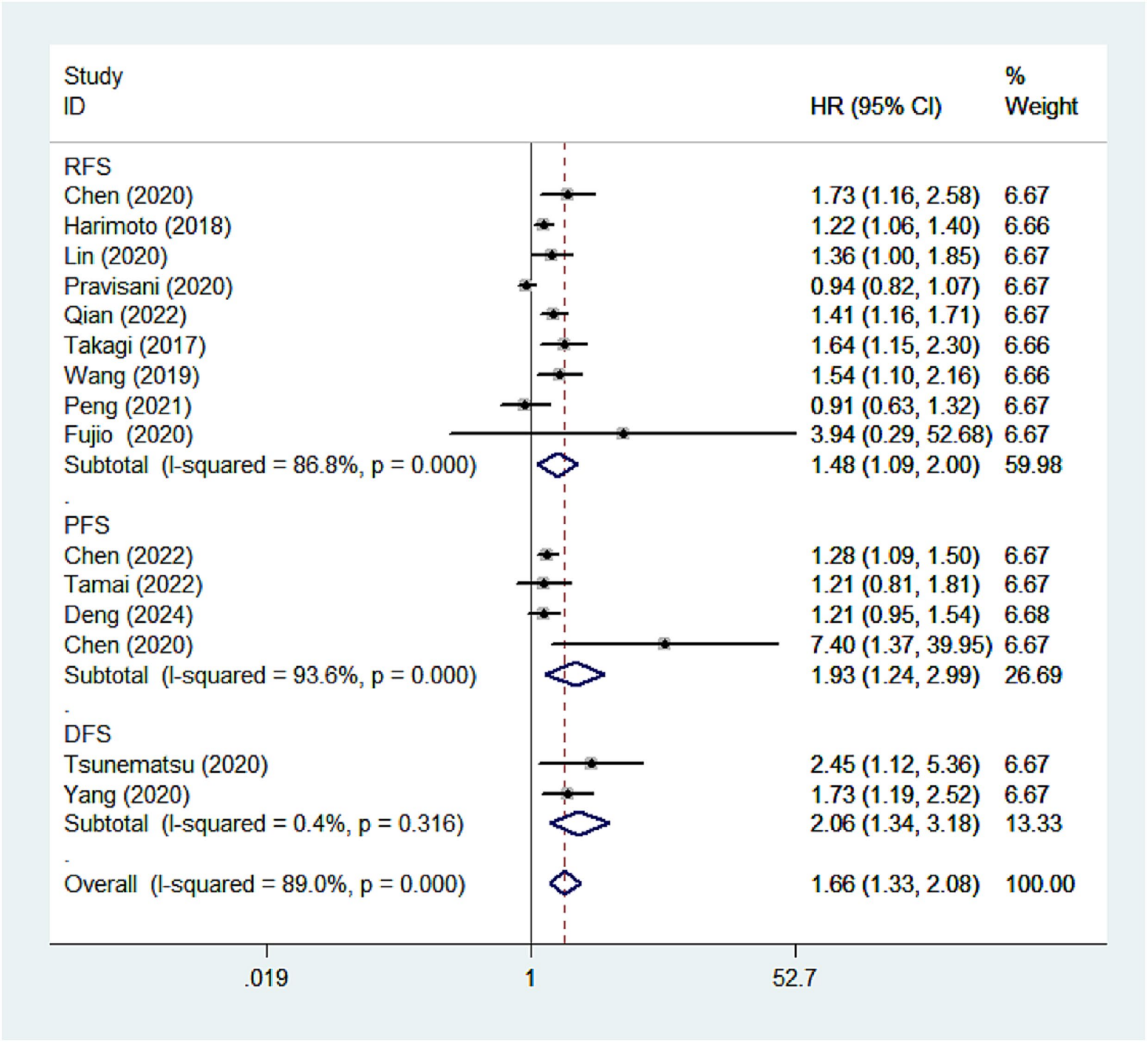
Forest plots of the association between high CONUT score and DFS/RFS/PFS. CONUT, controlling nutritional status; DFS/RFS/PFS, disease-free survival/recurrence-free survival/progression-free survival.

We further investigated the relationship between CONUT scores and different survival outcomes. Nine studies analyzed the association between CONUT scores and RFS, four studies explored the relationship between CONUT scores and PFS, and two studies assessed the correlation between CONUT scores and DFS. The results indicated that patients with high CONUT scores generally exhibited poorer DFS (HR: 2.06, 95 %CI: 1.34–3.18), RFS (HR: 1.48, 95% CI: 1.09–2.00), and PFS (HR: 1.93, 95% CI: 1.24–2.99), further underscoring the prognostic value of CONUT score (Figure 3).

Subgroup analysis for RFS by country, treatment methods, sample size, and analysis type was performed (Table 3). High CONUT score was mainly associated with adverse RFS in the subgroups of China (HR:1.37, 95% CI:1.20–1.56), Japan (HR: 1.28, 95%CI: 1.12–1.45), non-surgery (HR: 1.73, 95%CI:1.16–1.41), surgery (HR: 1.25, 95%CI: 1.05–1.47), sample size (>300) (HR:1.28, 95% CI:1.16–1.41), sample size (<300) (HR: 1.33, 95%CI:1.31–2.00) and multivariate analysis (HR: 1.35, 95% CI:1.23–1.49).

### Impact of CONUT score on clinicopathological features

To further evaluate the association between high CONUT score and clinicopathological features, the investigators collected relevant clinicopathological data (Table 4). No significant association was found between high CONUT scores and sex, age, or tumor diameter, indicating that these factors may have a limited influence on the CONUT score. However, a significant correlation was observed between high CONUT scores ad tumor differentiation (poor vs. moderate/well) (OR: 1.32, 95% CI: 1.16–2.35) as well as tumor number (solitary vs. multiple) (OR: 1.25, 95% CI: 1.19–1.84). Specifically, high CONUT score was generally associated with poorer tumor differentiation and a greater number of tumors, suggesting that CONUT score may be closely linked to the degree of malignancy and progression of HCC.

**Table 4:** Association between high CONUT score and clinicopathological features.

Clinicopathologic features	No. of studies	Estimate OR (95%CI)	*p*-value	Heterogeneity		
				I^2^ (%)	*p*-value	Model
Gender (male vs. female)	8	0.71 (0.52–2.12)	0.442	30.8	0.566	Fixed
Age (>60 vs. <60)	4	1.74 (0.61–5.31)	0.511	71	0.023	Random
Tumor diameter (Big vs. Small)	8	0.83 (0.61–1.33)	0.481	0	0.203	Fixed
Tumor differentiation (poor vs. moderate/well)	7	1.32 (1.16–2.35)	0.007	12.5	0.335	Fixed
Tumor number (solitary vs. multiple)	7	1.25 (1.19–1.84)	0.02	41	0.415	Fixed

### Sensitivity analysis

**Figure 4:**
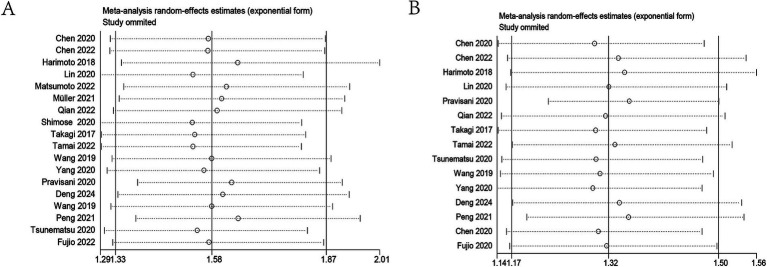
Sensitivity analysis. **(A)** Sensitivity analysis for OS. **(B)** Sensitivity analysis for DFS/RFS/PFS. OS, overall survival; DFS/RFS/PFS, disease-free survival/recurrence-free survival/progression-free survival.

Sensitivity analyses were conducted to evaluate the stability of the results (Figures 4A,B). By sequentially removing each study and rerunning the pooled analysis, the findings demonstrated that excluding any individual study did not significantly alter the pooled results. These results indicated that despite the inclusion of numerous studies from diverse regions, the overall analysis results remained robust and were not substantially influenced by any single study.

### Publication bias

**Figure 5:**
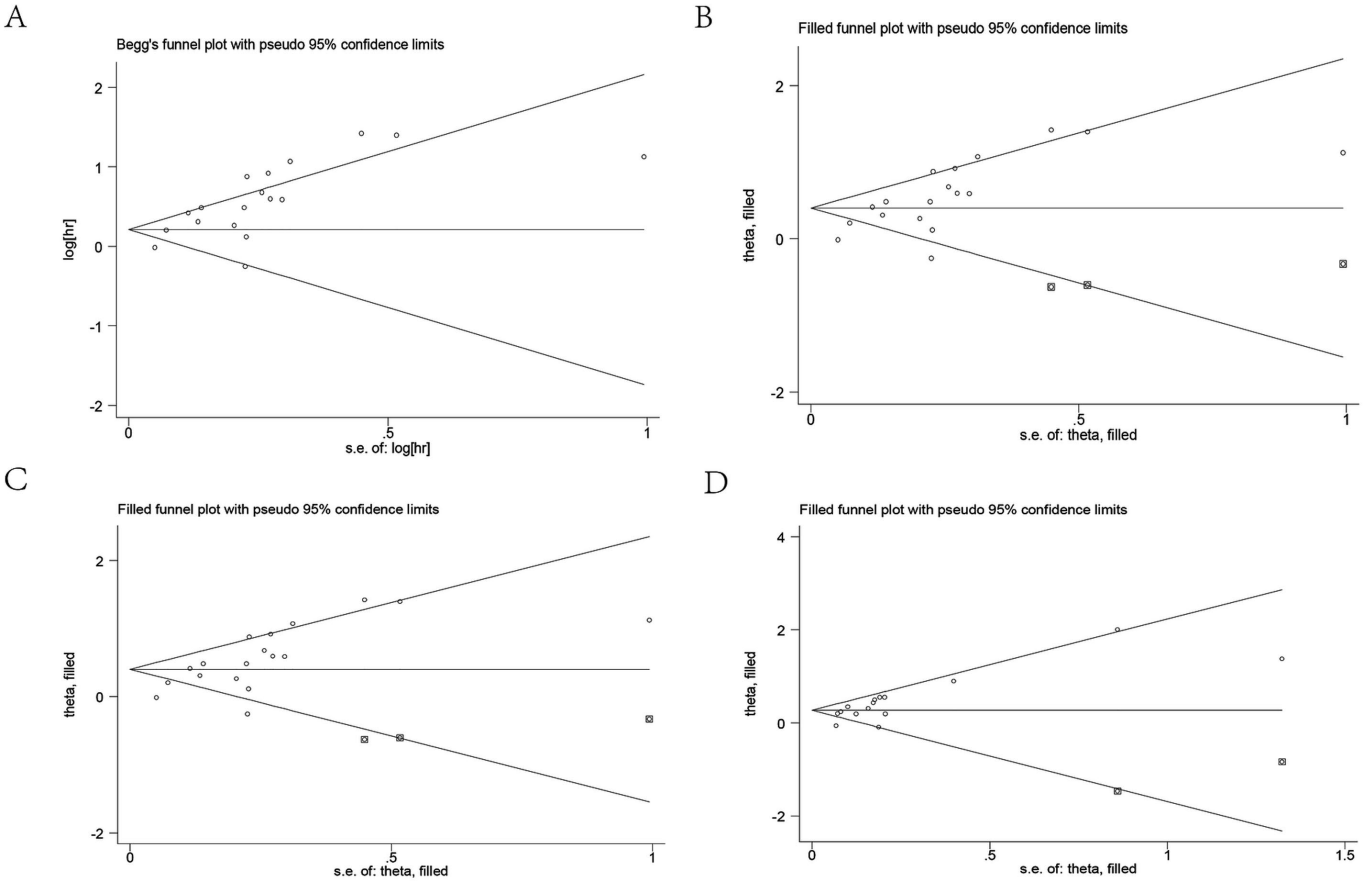
Publication bias. **(A)** Publication bias for OS. **(B)** Trim-and-fill method for OS. **(C)** Publication bias for DFS/RFS/PFS. **(D)** Trim-and-fill method for DFS/RFS/PFS. OS, overall survival; DFS/RFS/PFS, disease-free survival/recurrence-free survival/progression-free survival.

Publication bias in this study was assessed using Begg’s test and Egger’s test. The *p*-values of Begg’s test and Egger’s test for OS were 0.058 and 0.001, respectively. The results showed a publication bias for OS (Figure 5A). Therefore, we assessed the stability of the combined results using the trim-and-fill method, which showed that the result for OS was not affected (HR: 1.49, 95CI%: 1.26–1.77) (Figure 5B). The p-values of Begg’s test and Egger’s test for DFS/RFS/PFS were 0.018 and 0.009, respectively (Figure 5C). The trim-and-fill method also confirmed that the meta-analysis was not affected by bias (HR: 1.31, 95%CI: 1.152–1.49) (Figure 5D). The robustness of the conclusions should be interpreted with caution. This bias may reflect a tendency to publish studies with positive findings, potentially overestimating the true effect. While the statistical adjustment mitigated some of this influence, the implications of such bias cannot be fully excluded. Further high-quality, prospective studies were warranted to validate these findings.

## Discussion

The CONUT score is a widely utilized nutritional screening tool that effectively assesses the immune and nutritional status of patients. By evaluating albumin levels, lymphocyte counts, and cholesterol levels, it provides a comprehensive assessment of immune response and nutritional condition, making it a significant prognostic indicator. Several meta-analyses have confirmed that the CONUT score serves as a valuable tool for predicting patient prognosis (35, 36). Although the CONUT score has been extensively applied in prognostic assessments across various tumors, its specific prognostic value in HCC remains incompletely defined, necessitating further studies to explore and validate its utility.

A total of 19 studies involving 7,963 patients were included in this meta-analysis. The studies encompassed data from different regions, sample sizes, and study designs. A comprehensive analysis revealed that patients with higher CONUT score had worse OS, DFS, RFS, and PFS. Subgroup analysis further confirmed the prognostic value of the CONUT score. Compared to previous meta-analyses, this study included a larger number of studies and patients. By expanding the sample size and incorporating subgroup analysis, this study provides stronger evidence to support the clinical application of the CONUT score. Compared with other indicators, such as the neutrophil-to-lymphocyte ratio (NLR), platelet-to-lymphocyte ratio (PLR), and prognostic nutrition index (PNI), the CONUT score has higher predictive accuracy (37). Although multiple studies have investigated the prognostic value of the CONUT score in HCC, the limited sample sizes in individual studies have raised concerns regarding the reliability of their conclusions. By synthesizing data from multiple cohorts, our meta-analysis provided a more robust and comprehensive assessment, confirming that the CONUT score was a valuable prognostic indicator in HCC patients. Given the liver’s central role in metabolism and immunity, these abnormalities are frequently observed in HCC patients and are associated with poor prognosis. Therefore, the CONUT score may offer additional prognostic value beyond conventional staging systems such as BCLC and Child-Pugh by capturing aspects of the host’s systemic condition not fully reflected in tumor burden or liver function scores. Its simplicity and objectivity also make it a potentially practical tool to guide treatment decision-making and risk stratification in clinical settings.

The CONUT score has been demonstrated to effectively predict the prognosis of patients with tumors by evaluating their nutritional and immune status. However, the specific mechanisms through which the CONUT score influences tumor prognosis remain incompletely understood, and in-depth biological studies are still lacking. To explore this phenomenon, we attempted to uncover the potential prognostic mechanisms by analyzing the components of the CONUT score, which includes serum albumin, total cholesterol levels, and lymphocyte count. These components are closely associated with immune response, nutritional status, and chronic inflammation, and may collectively contribute to the prognosis of patients with tumors.

The CONUT score incorporates three key components—serum albumin, total cholesterol, and lymphocyte count, which are closely associated with liver-specific pathophysiological processes. In patients with liver cirrhosis or HCC, hypoalbuminemia is commonly observed, indicating impaired liver function, malnutrition, systemic inflammation and immunosuppression (38–40). Lymphocyte count serves as a direct marker of immune competence. Lymphopenia suggests weakened cell-mediated immunity, which may compromise effective tumor immune surveillance and facilitate the progression of HCC (41–43). Similarly, reduced cholesterol levels often reflect poor nutritional status and diminished bile acid synthesis, both of which are prevalent in advanced liver disease. Beyond its function in cell membranes, cholesterol is also involved in inflammatory and immune responses, influencing the immune system’s functionality (44). Low cholesterol levels can impair the integrity and function of immune cell membranes, thereby weakening the body’s immune response against tumors (45–47). Studies have demonstrated that reduced levels of albumin, lymphocytes, and cholesterol are closely associated with poor prognosis in various malignancies, including HCC (48–53). These biomarkers capture the intertwined effects of malnutrition, inflammation, and immune dysregulation in the progression of HCC, highlighting the CONUT score as a relevant tool for risk stratification in this population. The specific mechanisms underlying the role of the CONUT score in hepatocellular carcinoma (HCC) remain to be fully elucidated. Emerging spatial multi-omics techniques offer promising tools to deepen our understanding of how the CONUT score reflects the complex interplay of nutritional status, immune function, and tumor microenvironment in HCC (54–56).

The high CONUT score is characterized by low levels of albumin, lymphocyte count, and cholesterol, which together form a comprehensive evaluation system. These indicators not only independently reflect patients’ nutritional status and immune function but also interrelate, jointly revealing the deterioration of overall health in cancer patients. Collectively, these factors underscore the CONUT score’s crucial role in predicting poor prognosis in HCC patients. Malnutrition and impaired immune function are not only risk factors for tumor progression but also may affect a patient’s tolerance to and recovery from treatment. By integrating these key indicators, the CONUT score offers a more comprehensive assessment of patients’ prognostic risk and provides an essential foundation for clinical decision-making.

The main limitations of the meta-analysis were as follows: Firstly, the majority of the included studies were retrospective, and this design may introduce bias, potentially affecting the accuracy and generalizability of the findings. Secondly, most of the studies were from Asia, with a notable absence of data from other regions and countries, which limited the external validity and generalizability of the results. Thirdly, the lack of standardized cut-off values for the CONUT score may affect the reproducibility and comparability of the results. Fourth, the reference values of CONUT score adopted in this study were set according to the common standards, and the impact of HCC on metabolism was not considered when setting the reference values. Finally, the presence of publication bias warranted cautious interpretation of the results, and further high-quality studies were needed to validate our conclusions.

The strengths of this meta-analysis were reflected in several key aspects. Firstly, it further confirmed the critical role of the CONUT score in the prognostic evaluation of HCC patients through the integration and analysis of large-scale data. Secondly, the study validated the prognostic value of the CONUT score across various clinical conditions through subgroup analysis. Thirdly, the results of the meta-analysis remained stable even after the removal of any individual study, demonstrating a high degree of reliability in the study’s conclusions. Finally, using the pruning and filling method, this study verified that publication bias did not significantly affect the meta-analysis results.

In conclusion, high CONUT score were significantly associated with poorer survival outcomes in patients with HCC. The CONUT score may serve as an effective prognostic indicator for evaluating the clinical outcomes of HCC patients. Clinicians can evaluate the nutritional status, immune function, and prognosis of HCC patients through the CONUT score and provide guidance for timely clinical treatment. Due to the some limitations, well-designed large-scale randomized controlled trials were needed to further verify our findings before large-scale clinical application.

## Data Availability

The original contributions presented in the study are included in the article/supplementary material, further inquiries can be directed to the corresponding authors.
